# Feasibility study demonstrating that enzymatic template generation and amplification can be employed as a novel method for molecular antimicrobial susceptibility testing

**DOI:** 10.1186/1471-2180-13-191

**Published:** 2013-08-13

**Authors:** Bruce I Sodowich, Daniel R Zweitzig, Nichol M Riccardello, S Mark O’Hara

**Affiliations:** 1 Zeus Scientific Incorporated, Research and Development, 200 Evans Way, Branchburg, NJ 08876, USA

## Abstract

**Background:**

Antimicrobial Susceptibility Testing (AST) is a methodology in which the sensitivity of a microorganism is determined via its inability to proliferate in the presence of an antimicrobial agent. Results are reported as minimum inhibitory concentrations (MICs). The present study demonstrates that measurement of DNA polymerase activity via Enzymatic Template Generation and Amplification (ETGA) can be used as a novel means of determining the MIC of a microbe to an antibiotic agent much sooner than the current standardized method.

**Methods:**

Time course analysis of ETGA is presented from bacterial cultures containing antibiotic agents and compared to the end-point results of standard macrobroth method AST.

**Results:**

MIC determinations from ETGA results at 4, 6, and 22 hours are compared to the MICs from the standard method and the results are shown to be in agreement. Additionally, reliable AST analysis using ETGA can be performed on bacteria harvested directly from spiked blood cultures.

**Conclusions:**

AST analysis with ETGA is shown to be equivalent to AST analysis using gene-specific qPCR assays against the measured microbe. Future development of this novel method for performing AST in a clinical setting is discussed.

## Background

Antimicrobial Susceptibility Testing (AST) is a method used to predict the response of a clinically isolated microorganism to antimicrobial agents so that the most appropriate therapy may be administered to a patient [1,2]. Typically, the results of AST are reported as minimum inhibitory concentrations (MICs), which is the minimum concentration of a particular agent that will inhibit the visible growth of a microorganism after overnight incubation [3]. AST can be performed in several ways, via disk diffusion or Kirby-Baur method [4,5], agar dilution, or broth dilution [6,7]. The sensitivity or resistance of an organism to a drug is based on the interpretation of the MIC compared to interpretive standards [8].

AST is routinely performed from positive blood cultures bottles from patients where bacteremia or sepsis is suspected. However, traditional methods of determining the AST profile may take up to 24 hours, and that does not include the additional time of 24–48 hours required for the isolation of the organism [9]. Therefore, reducing the time to results of AST on which physicians can make sound clinical decisions for the management of their patients would have both a significant positive clinical impact and be more cost effective [10,11]. Automated AST systems are currently available within the clinical diagnostics market [12], and the technology used by these platforms require bacterial isolation. However, several reports using automated AST systems have been published which indicate that reliable AST results can be achieved directly from the positive blood culture or with minimal sample preparation, which bypasses the need of time consuming bacterial isolation, and further reducing the time to results [13-15].

Recent literature has introduced the emerging technology of molecular AST [16-19] in which quantitative PCR is used to monitor the growth of bacterial cultures in the presence of antibiotic agents. They are based on the amplification of the *rpoB* gene; the 16S ribosomal locus universally found in the bacterial genome. The technology is based on the premise that the growth kinetics of bacteria in culture can be monitored by measuring the increasing amounts genomic DNA. In this fashion, MICs may be determined on the same day as the initial inoculation rather than an overnight incubation. The kinetics of increasing PCR signal from a growing culture in the presence of an antibiotic can be used to determine whether a pathogen is resistant or susceptible to the agent. Furthermore, one group reports a workflow in which molecular AST can be performed on bacteria harvested directly from blood culture using serum separation tubes, identifying the pathogen with species specific qPCR probes, and producing a molecular AST result in a single day [20].

Our group has previously reported a novel methodology termed Enzyme Template Generation and Amplification (ETGA) that enables universal, sensitive and quantitative measurement of bacterial proliferation via measurement of endogenous DNA polymerase activity [21]. In this report, we demonstrate that molecular AST and MIC determination can be performed via ETGA-mediated monitoring of DNA polymerase activity. We compare the functionality of ETGA AST to PCR-based molecular AST using gene-specific qPCR assays (gsPCR) against either *S. aureus* or *E. coli*. We also show that ETGA AST can be used to determine MICs from bacteria harvested directly from spiked blood cultures.

## Methods

### Bacterial strains, cultivation, and antibiotics tested

The following strains were used in this study: *Escherichia coli* ATCC 25922, methicillin susceptible *Staphylococcus aureus* ATCC 29213, and methicillin resistant *Staphylococcus aureus* NRS241. All strains were propagated on Brain-Heart Infusion Agar (Teknova, Hollister, CA). The S. *aureus* strains, both methicillin resistant and susceptible, were tested for susceptibility against oxacillin and vancomycin (Sigma Aldrich, St. Louis, MO). The *E. coli* strain was tested for susceptibility against ciprofloxacin and tetracycline (Sigma Aldrich, St. Louis, MO).

### Macrodilution broth method for the determination of antimicrobial susceptibility

The macrobroth dilution method and the interpretive standards for determining the antimicrobial susceptibility of a microorganism to an antimicrobial agent are published by the Clinical and Laboratory Standards Institute [6,8]. In brief, an antibiotic was diluted into 2 mL of Cation-Adjusted Mueller Hinton Broth (CA-MHB, Teknova, Hollister, CA) at twice the concentration of the highest level of agent that will be tested in a 14 mL polypropylene round-bottom tube (Becton-Dickenson, Franklin Lakes, NJ). This was serially diluted in two-fold steps (1 mL: 1 mL) to create the desired antibiotic range; each tube containing twice the ultimate concentration of drug in 1 mL of broth. An additional tube containing 1 mL of broth without drug is also prepared as the growth control. For testing *S. aureus*, the CA-MHB was supplemented with additional NaCl to a final concentration of 2% (w/v) in order to enhance the methicillin resistant phenotype, if present, when testing for susceptibility against oxacillin [6,22]. Freshly grown colonies of the microorganism to be tested were suspended in a 0.9% saline solution and adjusted to a 0.5 McFarland standard. This bacterial suspension was further diluted in CA-MHB 1:150-fold and 1 mL of this secondary suspension was added to each broth containing antibiotic. This produces a series of 2 mL cultures containing the desired range of antibiotic in which each culture contains approximately 5.0E + 05 CFU/mL of bacteria. The inoculation concentration was verified by removing a 0.01 mL aliquot from the growth control culture, diluting it 1000-fold in 0.9% saline solution and directly plating 0.1 mL for CFU enumeration. The cultures were incubated at 35 ± 2°C, shaking at 350 rpm for 20–24 hours. The MIC of the drug/bacteria combination is determined as the culture containing the lowest concentration of antibiotic which fully inhibits the propagation of the culture (no visual turbidity) after the incubation period.

### Time course sampling of the AST cultures and ETGA substrate conversion

**Figure 1:**
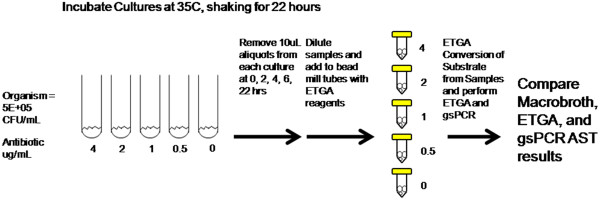
**Experimental design of the study.** On day one, the macrobroth AST is assembled. At the indicated time points, an aliquot is removed from each broth and diluted ten-fold. A portion of the diluted sample is subjected to bead milling for bacterial lysis, and incubated for ETGA substrate conversion. Once processed, the samples are stored at -20°C prior to analysis. On day two, the MIC of the AST is determined by visual turbidity. A final time point sample from each culture is taken and processed as day one, except the samples from any turbid cultures are diluted 1:10,000 fold. Once all samples are processed, the sample set is analyzed through the qPCR readout portion of the assay. These samples are also analyzed using the appropriate gene-specific qPCR assay as a comparison. The MIC as determined by the molecular AST analyses were compared to the MIC as determined from the predicate macrobroth analysis to determine the agreement between these methods.

The experimental design of the study is shown in Figure 1. After inoculation of each macrodilution broth with approximately 5.0E + 05 CFU/mL of bacteria, at 0, 2, 4, 6, and 22 hours (the overnight incubation) a 0.01 mL aliquot was removed from each culture and diluted 1:10 in nuclease free water (Life Technologies, Carlsbad, CA). If the sample was taken from a turbid culture after 22 hours of incubation, the sample was diluted 1:1E + 04 in nuclease free water by serial dilution. From each diluted sample, 0.01 mL was removed and placed into a 1.5 mL screw-capped tube containing glass beads and 0.05 mL of ETGA reaction solution. The bead-mill tubes were subsequently milled for bacteria lysis, incubated at 37°C for 20 minutes followed by 95°C for 5 minutes (to terminate the reaction), spun down, and stored at -20°C prior to analysis. At the final time point, ETGA reagent and positive controls [21] were performed alongside the samples.

A brief description of the mechanism of the ETGA assay is as follows; the ETGA reaction solution bead mill tube is formulated to facilitate microbe-derived DNA polymerase-mediated extension of a primer-template oligonucleotide substrate. Upon bead milling, microbe cell wall lysis allows contact between active microbe derived DNA polymerases and the primer-template substrate. A successful DNA polymerase primer-template extension event of the substrate’s primer oligonucleotide provides a new primer binding site for a subsequent qPCR detection reaction. Thus, DNA polymerase extension activity enables and triggers a downstream qPCR detection reaction. The subsequent qPCR detection signal is directly proportional to the amount of substrate extended, which is proportional of the amount of microbial DNA polymerase extension activity present, and this is proportional to the amount of viable proliferating bacteria present from culture. Complete details regarding the ETGA assay have been previously described [21] a hyperlink is provided [http://nar.oxfordjournals.org/content/40/14/e109.full.pdf+html?sid=ea56a354-4e91-4515-aec8-ccdc5acfb438].

### ETGA and gene-specific qPCR analysis of the time course samples

Stored samples were allowed to thaw at room temperature, briefly vortexed, and spun down at 12,000×g for one minute. ETGA readout by qPCR was performed by adding 4 μL of each sample into a reaction well containing 27.2 μL of qPCR reaction mix which has been previously described [21].

For the parallel-run of corresponding gsPCR for either *S. aureus* or *E. coli* samples, single reactions were run composed of 3 μL bead mill lysate added to 28 μL of the appropriate qPCR reaction mix into a reaction well. The gene targets for the *S. aureus* and *E. coli*-specific qPCR assays are *nuc* and *uidA* respectively. The primer and probe sequences for these assays have been previously reported [21]. All qPCR analysis was performed on a Roche LightCycler 480 II system (Roche Applied Science, Indianapolis, IN). Cycle values were plotted against time of incubation. The values produced by the overnight samples were plotted as the measured Ct minus 10 to account for the 1000-fold dilution compared to the earlier samples. This assumes that each 10-fold dilution equates to a 3.33 cycle decrease in signal based on an efficient qPCR reaction.

### ETGA AST analysis from bacteria harvested from a BD BACTEC positive blood culture bottle

Institutional review board approval of our procedure and consent form was provided by the Essex Institutional Review Board Inc. 121 Main St. Lebanon NJ. Eight to 10 mL of blood from consenting healthy donors were collected into a BACTEC Plus + Aerobic/F bottle BD, Franklin Lakes, NJ). This blood culture was then spiked with 5 to 50 CFU of either *S. aureus* (MSSA or MRSA) or *E. coli* bacteria. The blood culture bottle was incubated in a BD BACTEC 9050 incubator and grown until the culture is called positive. Once positive, the bacteria were harvested with a Serum Separation Tube (SST) (BD, Franklin Lakes, NJ) as described elsewhere [19,20]. Briefly, the tube was spun for 10 minutes at 2000×g and the supernatant was removed. A sterile, rayon-tipped swab applicator (BD, Franklin Lakes, NJ) was used to harvest the bacteria from the gel layer of the tube and this was suspended into a 0.9% saline solution. From this point forward, these SST preparations were handled the same as described for pure cultures, except time points were only taken at four and six hours of incubation.

### Comparison of molecular AST results to the marcobroth “gold standard” method results

The macrobroth method results are considered the “gold standard” results because they are performed based on the currently accepted method as indicated by CLSI documentation. Differences between the molecular AST results and the gold standard results are defined as follows: 1) an error is called minor when the molecular AST indicates susceptibility and the macrobroth AST indicates intermediate resistance, 2) an error is called major when the molecular AST indicates resistance and the macrobroth AST indicates susceptibility, and 3) an error is called very major when the molecular AST indicates susceptibility and the macrobroth method indicates resistance [12].

### Additional data sets

**Figure 2:**
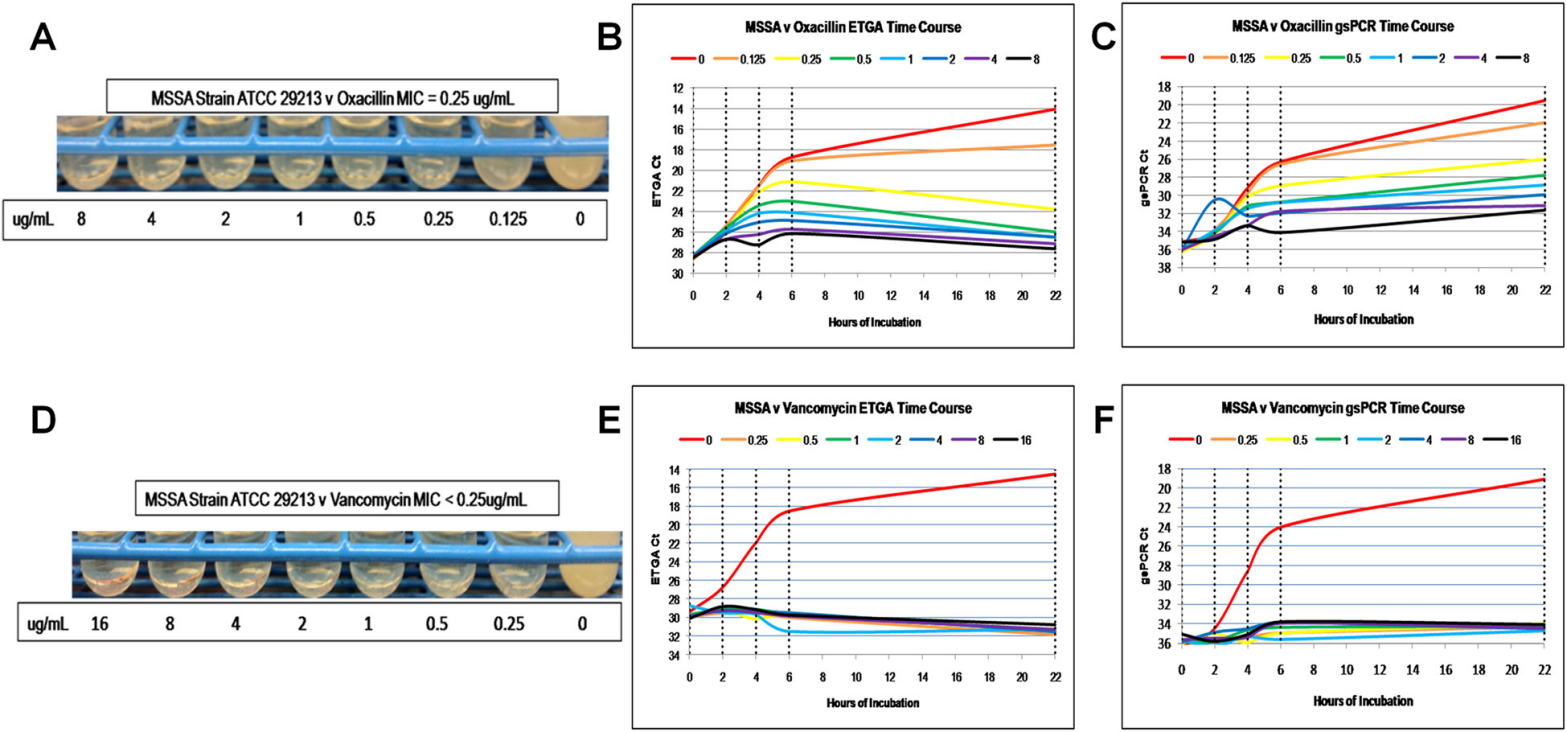
**Methicllin sensitive *Staphylococcus aureus *against oxacillin and vancomycin AST results.** The visual results of the macrobroth dilution standard method is shown on the left **(A and D)**, along with the time course results of the ETGA **(B and E)** and gsPCR **(C and F)** AST analyses, plotting Ct versus time. Vertical, dashed lines indicate when aliquots were removed for analysis. Since Ct values are inversely related to signal strength, the y-axes are inverted to visually demonstrate a rise in signal over time.

**Figure 3:**
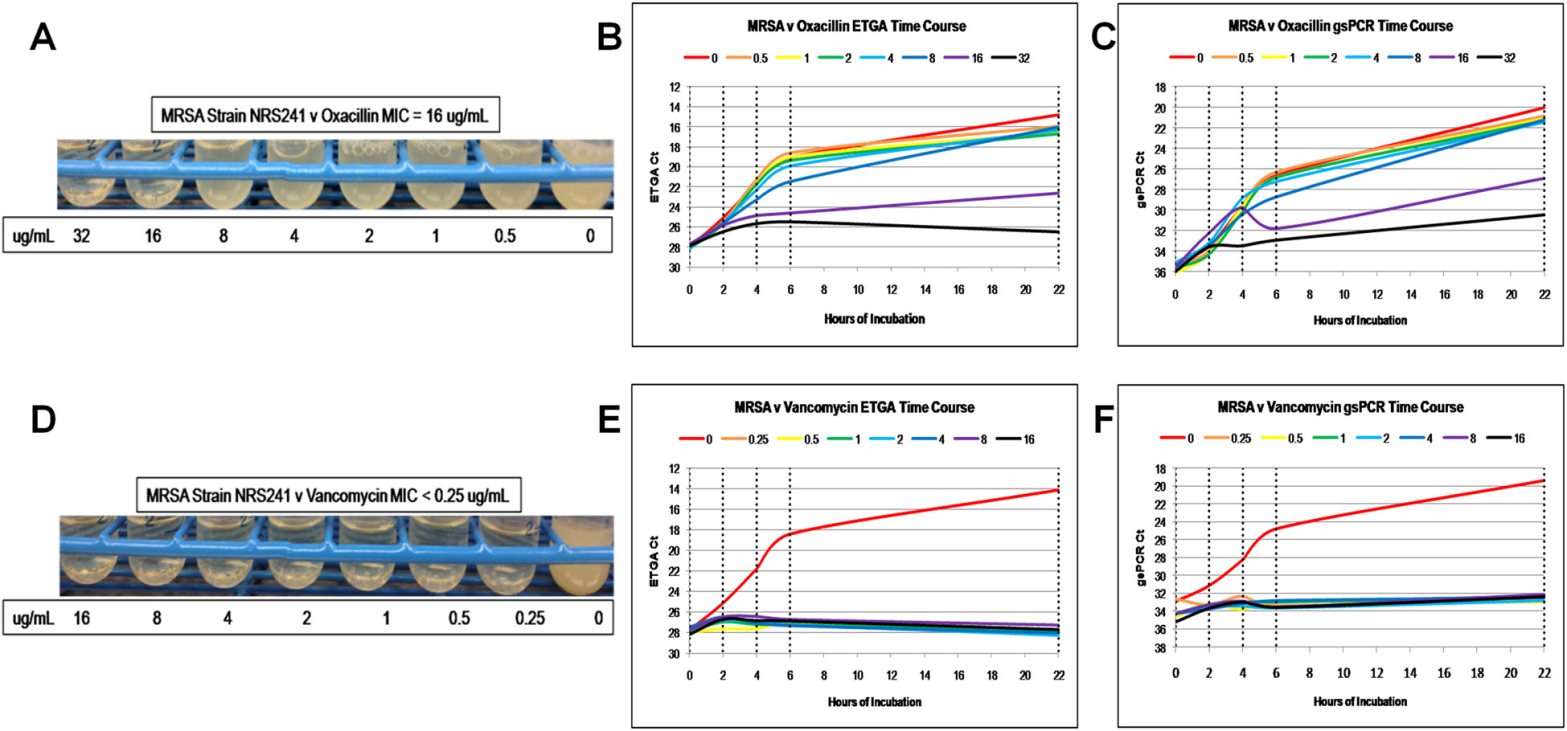
**Methicillin resistant *Staphylococcus aureus *against oxacillin and vancomycin AST results.** The visual results of the macrobroth dilution standard method is shown on the left **(A and D)**, along with the time course results of the ETGA **(B and E)** and gsPCR **(C and F)** AST analyses, plotting Ct versus time. Vertical, dashed lines indicate when aliquots were removed for analysis. Since Ct values are inversely related to signal strength, the y-axes are inverted to visually demonstrate a rise in signal over time.

**Figure 4:**
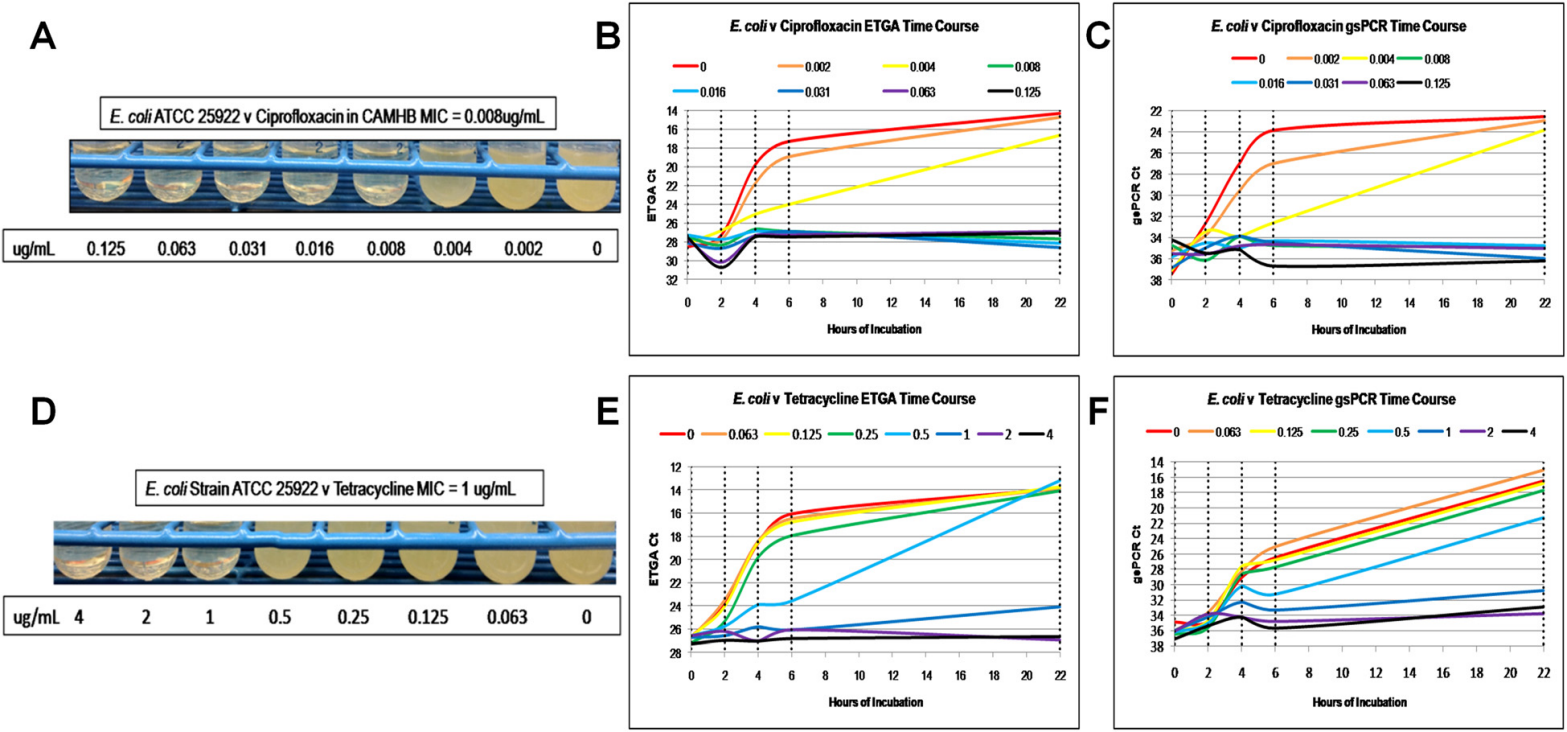
***E. coli *against ciprofloxacin and tetracycline AST results.** The visual results of the macrobroth dilution standard method is shown on the left **(A and D)**, along with the time course results of the ETGA **(B and E)** and gsPCR **(C and F)** AST analyses, plotting Ct versus time. Vertical, dashed lines indicate when aliquots were removed for analysis. Since Ct values are inversely related to signal strength, the y-axes are inverted to visually demonstrate a rise in signal over time.

Additional data sets are provided which detail all the cycle time data used to produce figure and data found within this manuscript. The file in which these data can be found is called Supplemental Data to manuscript.doc. Within this file is Additional file 1: Table S1 and Additional file 1: Table S2. Additional file 1: Table S1, ETGA and gsPCR Ct Data of AST Experiments from Pure Cultures, provides data used for Figures 2, 3, and 4 and pure culture data in Table 1. Additional file 1: Table S2, ETGA and gsPCR Ct Data of AST Experiments from Cultures Harvested from Positive Blood Cultures, provides data for the AST experiments from bacteria harvested from blood culture found in Table 1.

**Table 1:** **Comparison of minimum inhibitory concentration results for MSSA, MRSA and *E. coli *strains**


***S. aureus ATCC 29213***													
				**Bacteria from purified cultures**						**Bacteria harvested from a positive blood culture bottle**			
**Drug**	**Phenotype**	**CLSI MIC interpretation**	**Macrobroth MIC and interpretation**	**ETGA MIC and interpretation**			**gsPCR MIC and interpretation**			**ETGA MIC and interpretation**		**gsPCR MIC and interpretation**	
				4 hr	6 hr	22 hr	4 hr	6 hr	22 hr	4 hr	6 hr	4 hr	6 hr
Oxacillin	Susceptible	S ≤ 2 R ≥ 4	0.25 S	1 S	0.5 S	0.5 S	0.5 S	1 S	1 S	0.25 S	0.25 S	0.25 S	0.25 S
Vancomycin	Susceptible	S ≤ 2 I = 4-8 R ≥ 16	< 0.25 S	<0.25 S	<0.25 S	<0.25 S	<0.25 S	<0.25 S	<0.25 S	<0.125 S	<0.125 S	<0.125 S	<0.125 S
***S. aureus NRS241***													
				**Bacteria from purified cultures**						**Bacteria harvested from a positive blood culture bottle**			
**Drug**	**Phenotype**	**CLSI MIC interpretation**	**Macrobroth MIC and interpretation**	**ETGA MIC and interpretation**			**gsPCR MIC and interpretation**			**ETGA MIC and interpretation**		**gsPCR MIC and interpretation**	
				4 hr	6 hr	22 hr	4 hr	6 hr	22 hr	4 hr	6 hr	4 hr	6 hr
Oxacillin	Resistant	S ≤ 2 R ≥ 4	16 R	8 R	16 R	> 16 R	N/A^a^	16 R	>16 R	8 R	16 R	8 R	2 S^c^ (VME)
Vancomycin	Susceptible	S ≤ 2 I = 4-8 R ≥ 16	< 0.25 S	<0.25 S	<0.25 S	<0.25 S	<0.25 S	<0.25 S	<0.25 S	<0.25^b^ S	<0.25 S	<0.25^d^ S	<0.25^d^ S
***E. coli ATCC 25922***													
				**Bacteria from purified cultures**						**Bacteria harvested from a positive blood culture bottle**			
**Drug**	**Phenotype**	**CLSI MIC interpretation**	**Macrobroth MIC and interpretation**	**ETGA MIC and interpretation**			**gsPCR MIC and interpretation**			**ETGA MIC and interpretation**		**gsPCR MIC and interpretation**	
				4 hr	6 hr	22 hr	4 hr	6 hr	22 hr	4 hr	6 hr	4 hr	6 hr
Ciprofloxacin	Susceptible	S ≤ 1 I = 2 R ≥ 4	0.008 S	0.016 S	0.016 S	0.031 S	0.016 S	0.016 S	0.031 S	0.008 S	0.008 S	0.004 S	0.008 S
Tetracycline	Susceptible	S ≤ 4 I = 8 R ≥ 16	1 S	0.5 S	0.5 S	1 S	1 S	1 S	1 S	0.5 S	0.5 S	0.25 S	0.5 S

All MIC values are in units of μg/mL. Interpretation of MIC values are *S* susceptible, *I* intermediate resistance, and *R* resistant. MICs are determined from the molecular assays as the culture with the lowest concentration of drug that produces a difference in Ct value that remains less than 3.33 cycles between its Ct value and the Ct value of the culture with the highest concentration of drug, where growth is fully inhibited. Four discrepancies are noted: ^a^At 4 hours, the MIC value of the gsPCR method of MRSA versus oxacillin could not be determined since the difference in Ct values moved above and below the cut-off value between several concentrations. ^b^At 4 hours, the MIC value of <0.25 μg/mL from the ETGA method of MRSA harvested from blood culture versus vancomycin is interpreted as susceptible and is in agreement with the macrobroth method. However, the 16 μg/mL culture from the AST series produced a Ct value that indicates resistance. ^c^At 6 hours, the MIC value of the gsPCR method of MRSA harvested from blood culture versus oxacilin is interpreted as susceptible, while the macrobroth method MIC is interpreted as resistant. This is defined as a very major error (VME). ^d^The gsPCR results from the MRSA harvested from blood culture versus vancomycin produced several reactions with negative results. The baseline was arbitrarily adjusted to account for the lack of signal for these reactions. All discrepancies are discussed in the text.

## Results

### Molecular AST time course analysis of bacteria from purified cultures

Methicillin sensitive *S. aureus* strain ATCC 29213 and *E. coli* strain ATCC 25922 are both quality control strains for the macrobroth method and estimated MICs for these organisms for the antibiotics tested against them are indicated by the CLSI protocols and standards [6]. The ranges of antibiotic concentrations that were tested are based upon these published values. Methicillin resistant *S. aureus* strain NRS241 has MICs against specific drugs published on the NARSA website (http://www.narsa.net) and the concentration range tested was based upon these values. The time course curves for both the ETGA and gsPCR molecular analysis is shown in Figures 2, 3 and 4 and compared to the visual end-point analysis of the macrobroth dilution method. The data sets containing the measured Ct values can be found in Additional file 1: Table S1.

The ETGA time course analysis for each antibiotic/microorganism combination tested demonstrate that in growth control cultures which contain no drug the ETGA signal increases robustly over time. Depending on the combination tested, however, the rate of change in signal depends on the amount of antibiotic present. For instance, the MSSA versus oxacillin combination (Figure 2B) shows that there is an increase in signal in the early time points out to 2 μg/mL, but the 22 hour time point only the 0 and 0.125 μg/mL cultures demonstrate a continuous increase in signal. At 22 hours, the curves actually indicate a decrease in signal from 0.25 to 8 μg/mL. These results correlate with the macrobroth results (Figure 2A), in which turbidity is seen only in the 0 and 0.125 μg/mL cultures. Using the gsPCR assay, the signals from all cultures increase over time (Figure 2C), although the rate slows as the concentration of antibiotic increases. The MSSA versus vancomycin time course analysis indicates that no antibiotic concentration beyond the growth control exhibits any increase in signal over time for either the ETGA or gsPCR assay. The vancomycin macrobroth dilution results are in agreement with the time course results (Figure 2D-2F).

The ETGA time course for MRSA versus oxacillin demonstrates an increase of signal over time out to 8 μg/mL, although the rate of growth appears to slow at 8 g/mL (Figure 3B). The macrobroth dilution results are in agreement with the ETGA curves, since turbidity is seen in all cultures out to 8 μg/mL (Figure 3A). The curves for 16 and 32 μg/mL tend to remain flat. Similar growth kinetics is observed using the gsPCR assay (Figure 3C), although the curves for all the concentrations trend upward. Identical to the MSSA versus vancomycin curves, no MRSA cultures other than the growth control displays turbidity or an increase of signal over time using either assay (Figure 3D-F).

The *E. coli* versus ciprofloxacin ETGA time course curves demonstrate growth from 0 to 0.004 μg/mL, with slower growth at 0.004 μg/mL (Figure 4B). Higher drug concentrations produce flat curves. This result is in full agreement with the macrobroth dilution results and the gsPCR growth curve results (Figure 4A and 4C). Against tetracycline, *E. coli* displays a robust ETGA signal increase over time out to 0.5 μg/mL (Figure 4E). The macrobroth results agree with the ETGA results by showing turbidity up to 0.5 μg/mL (Figure 4D). At 1 μg/mL and above, the cultures are clear. The time course analysis using the gsPCR assay is in agreement with both the ETGA time course results and the macrobroth results (Figure 4F).

### Molecular AST MIC determination of bacteria from purified cultures

Using the data collected from these time course analyses, the MIC for each antibiotic/microorganism combination was determined at 4, 6, and 22 hours, using both ETGA and gsPCR data. Each MIC was determined by comparing the difference in Ct between the culture with the highest concentration of antibiotic to each of the other cultures in the series. A difference in Ct of 3.33 or more (a 1 log difference in signal) indicates a reliable increase in signal and the culture is considered to be actively proliferating. Therefore, the lowest concentration of drug in which the difference in Ct value remains less than 3.33 cycles is called the MIC for that series.

The molecular MICs for each series were determined and compared to the macrobroth method as shown in Table 1. While the ETGA-determined MIC may differ by one or two-fold concentrations away from the macrobroth MIC, all series produced an ETGA MIC that was in agreement with the expected CLSI interpretation. This was the case at all time points. Similar results were obtained with gsPCR method, except at the 4 hour time point of the MRSA versus oxacillin series (Table 1, superscript a). At this time point the signal moved both above and below the 3.33 cycle breakpoint at several dilutions of drug, and a MIC was unable to be determined. These results provide evidence that ETGA can be used to generate a reliable MIC for AST analysis by as much as 16 hours sooner than traditional AST methods, and functions in a similar fashion to molecular AST analysis using gsPCR assays.

### Molecular AST MIC determination of bacteria from positive blood cultures

Beuving and colleagues [19,20] have demonstrated that molecular AST can be performed on bacteria harvested directly from positive blood cultures by collecting the microbes from the culture using a SST. Such a method could produce a reliable MIC for a series of antibiotics against a pathogenic microbe without the need for its isolation, thereby further reducing the time required to obtain a reliable result. The same methodology was applied to the following ETGA experiments. Blood cultures were spiked with MSSA, MRSA, or *E. coli*, allowed to be called positive in a BACTEC 9050 incubator, the bacteria were harvested with an SST, and molecular AST was performed as previously described in the materials and methods.

The results and comparison of the molecular analyses to the macrobroth dilution method are shown in Table 1 and Additional file 1: Table S2. Analysis was carried out as before using both molecular methods at the four and six hour incubation time points. ETGA analysis produced MIC values that were mostly in agreement with the macrobroth method and correlated with the CLSI interpretation. However, one discrepancy (Table 1, footnote b) was observed at the four hour time point of the MRSA versus vancomycin series. While the MIC was determined to be less than 0.25 μg/mL, the 16 μg/mL culture, produced a signal with a Ct value greater than 3.33 cycles above the baseline. This isolated result was neither supported by the results from the other cultures in the series, its paired gsPCR reactions, nor the results from the six hour time point. The result is most likely indicative of an operator error. Such a result can occur when performing standard AST dilution methods. CLSI and similar AST protocols provide guidelines for interpreting such results and repeating the testing, if necessary [6,7].

The gsPCR analysis produced similar results to the ETGA analysis (Table 1) with two important discrepancies that require attention. The first is MRSA versus oxacillin at the six hour time point (Table 1, superscript c). Using the gsPCR method, the MIC was called at 2 μg/mL. Based on CLSI interpretation, this MIC value represents a susceptible phenotype. The expected phenotype, however, is resistant, and this is verified by the macrobroth method, the ETGA method at four and six hours, and the gsPCR method at 4 hours. This result is considered to be a very major error, since the molecular result indicates susceptibility, but the predicate method indicates resistance. The difference in Ct value between the 32 μg/mL culture and 2 μg/mL culture is just below the 3.33 cycle cut-off. Had the MIC been called at 4 μg/mL, the result would have been in agreement.

The second discrepancy produced by the gsPCR method was in the series of MRSA versus Vancomycin (Table 1, superscript d). Many of the gsPCR reactions produced a negative result, particularly at the zero hour time point. The baseline was accounted for by giving an arbitrary Ct value to each of these reactions of 38, the approximate cycle time a single copy of gene target is detected by qPCR. Once the baseline was adjusted reliable results were obtained. When either sensitive or resistant S. *aureus* was harvested from the blood culture using the SST, the inoculation verification produced CFU counts that were too low to be enumerable. Unlike the gsPCR assay, the ETGA assay detected the presence of bacteria in the cultures at the zero hour time point (Additional file 1: Table S1 and Additional file 1: Table S2).

## Discussion and conclusions

This report describes preliminary data for the use of ETGA as a rapid molecular method for producing reliable AST results. The results demonstrate that aliquots of cultures in a two-fold dilution series of antibiotic can be removed and analyzed with ETGA to determine a MIC much sooner than visual endpoint analysis that requires an overnight incubation of the cultures. The results of ETGA AST also correlate well with molecular AST results using gsPCR assays.

Recent literature describes molecular AST methods that employ qPCR assays which amplify the *rpoB* gene of the 16S rDNA locus of the bacterial genome as the marker for bacterial proliferation in culture [16,19,20]. The rDNA region is used as a universal gene target because the region is well conserved across prokaryotes and therefore only a single assay need be designed and validated. While the frequency of organisms that cause bacteremia has been fairly well defined [23] the list is by no means exhaustive. These studies shows genuine promise for the use of molecular AST as a method for achieving more rapid time to results, but the *rpoB* locus as a gene target may also create limitations. The rDNA region still exhibits considerable sequence variations across species, and degenerate primers and probes are required in order to detect a wide range of microorganisms [24-26]. Universal rDNA primers, no matter how well designed and validated, are not be able to amplify every possible organism or do so with equal efficiency.

Contrary to existing ‘universal’ PCR methodologies, ETGA is a highly sensitive enzymatic assay, not a genetic assay. Instead of genomic DNA, ETGA monitors bacterial proliferation in culture via measurement of endogenous DNA polymerase extension activity. Due to the highly conserved biochemical activity of DNA polymerase, the ETGA approach is not constrained to limitations in gene sequence based assays.

The ETGA AST method was successful in producing MICs that were in agreement with results obtained from the macrobroth dilution method using bacteria harvested directly from positive blood cultures. In contrast, gsPCR was less successful: MRSA versus oxacillin produced a very major error at six hours, and MRSA versus vancomycin produced gsPCR reactions that were not always detected. A point of concern with these experiments is that the inoculation verification indicated that the bacterial input was much lower than 5E + 05 CFU/mL because the CFU were too dilute to be countable. It has been reported that a 0.5 McFarland standard, which is expected to be within 1-2E + 08 CFU/mL may be as low as 1E + 07 CFU/mL depending on the species being measured [3]. While this lower titer appeared not to affect the macrobroth or the ETGA results, it may have affected the gsPCR results.

The procedure for harvesting the bacteria with a SST was followed as described by Beuving et al. [19,20]. However, their manuscripts do not indicate whether the investigators verified their inoculation concentration performing their molecular AST assays. Harvesting bacteria with SST from positive blood cultures was previously described by Funke and Funke-Kissling [13] for gram negative rods, and by Lupetti et al. [14] for gram positive cocci. In these reports, gram negative rods were harvested by applying positive blood culture directly into an SST, but gram positive cocci were first incubated in a 0.01% final concentration of saponin. The report from Beuving et al. harvests bacteria through an SST without any pre-treatment regardless of the gram status. If pre-treatment of the blood culture before serum separation is required for a more efficient bacterial yield, particularly for gram positive cocci, this could be a reason for some of the errors that we observed. Furthermore, we noticed that transferring bacteria from the gel plug to the saline solution can also lead to transferring some of the gel which could lead to overestimating the turbidity of the 0.5 McFarland standard. This observation presents an opportunity to further improve the sample preparation for increased bacterial yield harvested directly from positive blood cultures and ultimately improve the accuracy of molecular AST.

Wiegard et al. [7] describe a microdilution AST method performed in a 96-well microtiter plate. The authors present a protocol in which a bacterium of interest is inoculated into a matrix of various antibiotics and concentrations. This plate is incubated for 16–20 hours prior to interpreting the results by visual observation. Utilizing this design, a high-throughput ETGA AST method could be developed. In this scenario, an AST matrix can be assembled (keeping a few wells available for the required ETGA controls), and allowed to incubate for four to six hours. At a single time point, samples could be removed using a multi-channel pipette and diluted in water in a second micro-titer plate maintaining the same matrix pattern. After dilution, samples could then be transferred to a third micro-titer plate containing the ETGA reaction mix and glass beads. There are several 96-well format sample millers or homogenizers on the market that could be utilized to vortex the plate. After milling the plate would then be incubated at 37°C to enable substrate conversion. The samples could then be transferred to a final PCR microwell plate containing the ETGA qPCR reagents for the readout on a real-time PCR thermocylcer. The original AST plate could be returned to the incubator to produce an overnight result for verification purposes, if desired. Throughput could be further increased and error rate further reduced by designing a robotic system for the workflow.

This report has demonstrated that ETGA-mediated monitoring of bacterial DNA polymerase activity can be used to perform molecular AST and produce a reliable susceptibility interpretation that is equivalent to the CLSI macrodilution method in approximately 6 hours instead of 20–24 hours. This method has an advantage over PCR-based molecular AST that uses a gene target as the analyte because it is more universal in nature. These results suggest that it is possible to perform ETGA AST on bacteria harvested directly from blood culture without the need for extensive isolation and subculture, further reducing the time to results. In future experiments, ETGA AST will be validated against a wider array of pathogenic microbes and antimicrobial agents. This will be done on both bacterial isolates and directly from clinical culture samples. Further development of ETGA AST as a method that can be used in a clinical laboratory setting is ongoing.

## Competing interest

Bruce Sodowich, Daniel Zweitzig, Nichol Riccardello, and S. Mark O’Hara are all employees of Zeus Scientific Incorporated, a medical diagnostics company.

## Authors’ contributions

BS designed and executed experiments, and drafted the manuscript. DZ provided technical and critical review of the experimental design and results, and edited the manuscript. NR provided necessary laboratory support and repeated experimentation as necessary. SOH is the group leader and principal investigator. All authors read and approved the final manuscript.

## Supplementary Material

Additional file 1: Tables S1 ETGA and gsPCR Ct data of AST experiments from pure cultures. Values in bold indicate the concentration in which the MIC was called. Values in red indicate discrepancies in the results. **Table S2**: ETGA and gsPCR Ct data of AST experiments from cultures harvested from positive blood cultures. Values in bold indicate the concentration in which the MIC was called. Values in red indicate discrepancies in the results. Click here for file
