# Supplementary material for: Feasibility study demonstrating that enzymatic template generation and amplification can be employed as a novel method for molecular antimicrobial susceptibility testing
Source: BMC Microbiol. 2013 Aug 13;13:191. doi: 10.1186/1471-2180-13-191 (PMC3766015; doi:10.1186/1471-2180-13-191)
Supplement: Additional file 1: Tables S1 — ETGA and gsPCR Ct data of AST experiments from pure cultures. Values in bold indicate the concentration in which the MIC was called. Values in red indicate discrepancies in the results. Table S2: ETGA and gsPCR Ct data of AST experiments from cultures harvested from positive blood cultures. Values in bold indicate the concentration in which the MIC was called. Values in red indicate discrepancies in the results. [file 1471-2180-13-191-S1.doc]

| **ETGA Ct of Purified MSSA v Concentration of Oxacillin in Culture Over Time** | | | | | | | | |
| --- | --- | --- | --- | --- | --- | --- | --- | --- |
| **hours** | 0 | 0.125 | 0.25 | 0.5 | 1 | 2 | 4 | 8 |
| 0 | 28.59 | 28.72 | 28.24 | 28.23 | 28.30 | 28.30 | 28.33 | 28.49 |
| 2 | 26.06 | 25.36 | 25.73 | 25.57 | 25.84 | 26.13 | 26.70 | 26.70 |
| 4 | 21.48 | 21.51 | 22.11 | 23.45 | 24.15 | 25.05 | 26.24 | 27.24 |
| 6 | 18.76 | 19.06 | 21.09 | 23.04 | 24.08 | 24.87 | 25.74 | 26.13 |
| 22 | 14.09 | 17.51 | 23.75 | 25.98 | 26.47 | 26.46 | 27.13 | 27.62 |
|  | **ETGA ΔCt from the 8ug/mL Culture** | | | | | | | |
| 4hr | 5.76 | 5.73 | 5.13 | 3.79 | **3.09** | 2.19 | 1.00 | 0.00 |
| 6hr | 7.37 | 7.07 | 5.04 | **3.09** | 2.05 | 1.26 | 0.39 | 0.00 |
| 22 hr | 13.53 | 10.11 | 3.87 | **1.64** | 1.15 | 1.16 | 0.49 | 0.00 |

**Supplemental Data Set 1.** ETGA and gsPCR Ct Data of AST Experiments from Pure Cultures. Values in bold indicate the concentration in which the MIC was called. Values in red indicate discrepancies in the results.

ETGA Reagent Control Ct: 36.74

Polymerase Control Ct: 26.58

ETGA Reagent Control Ct: 36.74

| **gsPCR Ct of Purified MSSA v Concentration of Oxacillin in Culture Over Time** | | | | | | | | |
| --- | --- | --- | --- | --- | --- | --- | --- | --- |
| **hours** | 0 | 0.125 | 0.25 | 0.5 | 1 | 2 | 4 | 8 |
| 0 | 35.20 | 35.89 | 36.27 | 36.01 | 35.98 | 36.05 | 36.02 | 35.21 |
| 2 | 34.09 | 33.82 | 34.31 | 34.15 | 33.92 | 30.54 | 34.52 | 34.89 |
| 4 | 29.15 | 29.77 | 30.17 | 31.17 | 31.49 | 32.32 | 33.31 | 33.35 |
| 6 | 26.27 | 26.56 | 28.93 | 30.74 | 30.83 | 31.99 | 31.75 | 34.14 |
| 22 | 19.46 | 21.94 | 26.00 | 27.78 | 28.90 | 29.97 | 31.12 | 31.66 |
|  | **gsPCR ΔCt from 8ug/mL of Culture** | | | | | | | |
| 4hr | 4.20 | 3.58 | 3.18 | **2.18** | 1.86 | 1.03 | 0.04 | 0.00 |
| 6hr | 7.87 | 7.58 | 5.21 | 3.40 | **3.31** | 2.15 | 2.39 | 0.00 |
| 22hr | 12.20 | 9.72 | 5.66 | 3.88 | **2.76** | 1.69 | 0.54 | 0.00 |

| **ETGA Ct of Purified MSSA v Concentration of Vancomycin (µg/mL) in Culture Over Time** | | | | | | | | |
| --- | --- | --- | --- | --- | --- | --- | --- | --- |
| **hours** | 0 | 0.25 | 0.5 | 1 | 2 | 4 | 8 | 16 |
| 0 | 29.42 | 29.92 | 29.80 | 29.69 | 28.76 | 29.86 | 29.93 | 30.15 |
| 2 | 26.77 | 29.49 | 29.27 | 29.08 | 29.52 | 29.31 | 29.22 | 28.85 |
| 4 | 21.97 | 29.46 | 30.14 | 29.12 | 29.69 | 29.46 | 29.45 | 29.18 |
| 6 | 18.56 | 29.99 | 29.74 | 29.56 | 31.54 | 29.50 | 29.86 | 29.76 |
| 22 | 14.57 | 31.84 | 31.45 | 31.53 | 31.31 | 31.54 | 31.32 | 30.83 |
|  |  |  |  |  |  |  |  |  |
| **ETGA ΔCt from the 16ug/mL Culture** | | | | | | | | |
|  | 0 | 0.25 | 0.5 | 1 | 2 | 4 | 8 | 16 |
| 4hr | 7.21 | **-0.28** | -0.96 | 0.06 | -0.51 | -0.28 | -0.27 | 0.00 |
| 6hr | 11.20 | **-0.23** | 0.02 | 0.20 | -1.78 | 0.26 | -0.1 | 0.00 |
| 22hr | 16.26 | **-1.01** | -0.62 | -0.70 | -0.48 | -0.71 | -0.49 | 0.00 |

ETGA Reagent Control Ct: 39.57

Polymerase Control: 28.36

Inoculation Verification of Cultures: 15 CFU

| **gsPCR Ct of Purified MSSA v Concentration of Vancomycin (µg/mL) in Culture Over Time** | | | | | | | | |
| --- | --- | --- | --- | --- | --- | --- | --- | --- |
| **hours** | 0 | 0.25 | 0.5 | 1 | 2 | 4 | 8 | 16 |
| 0 | 36.37 | 36.43 | 35.97 | 35.85 | 35.65 | 35.92 | 35.65 | 35.05 |
| 2 | 34.49 | 35.55 | 35.03 | 35.8 | 36.25 | 34.87 | 35.50 | 35.80 |
| 4 | 28.59 | 35.35 | 35.92 | 34.64 | 35.31 | 34.51 | 35.43 | 35.08 |
| 6 | 24.05 | 34.91 | 34.97 | 34.33 | 35.61 | 33.84 | 33.93 | 33.78 |
| 22 | 19.11 | 34.30 | 34.04 | 34.22 | 34.70 | 34.51 | 34.42 | 34.05 |
|  |  |  |  |  |  |  |  |  |
|  | **gsPCR ΔCt from the 16ug/mL Culture** | | | | | | | |
|  | 0 | 0.25 | 0.5 | 1 | 2 | 4 | 8 | 16 |
| 4hr | 6.49 | **-0.27** | -0.84 | 0.44 | -0.23 | 0.57 | -0.35 | 0.00 |
| 6hr | 9.73 | **-1.13** | -1.19 | -0.55 | -1.83 | -0.06 | -0.15 | 0.00 |
| 22hr | 14.94 | **-0.25** | 0.01 | -0.17 | -0.65 | -0.46 | -0.37 | 0.00 |

| **ETGA Ct of Purified MRSA v Concentration of Oxacillin (µg/mL) in Culture Over Time** | | | | | | | | |
| --- | --- | --- | --- | --- | --- | --- | --- | --- |
| **hours** | 0 | 0.5 | 1 | 2 | 4 | 8 | 16 | 32 |
| 0 | 28.03 | 27.80 | 27.94 | 28.14 | 28.08 | 28.00 | 27.72 | 27.83 |
| 2 | 25.11 | 25.47 | 25.64 | 25.67 | 25.52 | 25.63 | 25.85 | 26.44 |
| 4 | 21.68 | 21.29 | 21.66 | 21.74 | 22.32 | 23.27 | 24.88 | 25.64 |
| 6 | 19.08 | 18.64 | 19.03 | 19.37 | 19.92 | 21.48 | 24.62 | 25.46 |
| 22 | 14.80 | 15.98 | 16.54 | 16.70 | 16.34 | 16.05 | 22.65 | 26.46 |
|  |  |  |  |  |  |  |  |  |
|  | **ETGA ΔCt from the 32ug/mL Culture** | | | | | | | |
| 4hr | 3.96 | 4.35 | 3.98 | 3.90 | 3.32 | **2.37** | 0.76 | 0.00 |
| 6hr | 6.38 | 6.82 | 6.43 | 6.09 | 5.54 | 3.98 | **0.84** | 0.00 |
| 22hr | 11.66 | 10.48 | 9.92 | 9.76 | 10.12 | 10.41 | 3.81 | **0.00** |

ETGA Reagent Control Ct: 36.74

Polymerase Control: 26.58

Inoculation Verification of Cultures: 24 CFU

| **gsPCR Ct of Purified MRSA v Concentration of Oxacillin (µg/mL) in Culture Over Time** | | | | | | | | |
| --- | --- | --- | --- | --- | --- | --- | --- | --- |
| **hours** | 0 | 0.5 | 1 | 2 | 4 | 8 | 16 | 32 |
| 0 | 35.29 | 35.18 | 36.15 | 35.63 | 35.13 | 35.65 | 35.54 | 35.97 |
| 2 | 33.57 | 34.03 | 34.26 | 34.32 | 33.20 | 33.47 | 32.22 | 33.57 |
| 4 | 29.69 | 29.57 | 29.68 | 30.20 | 28.85 | 30.45 | 29.74 | 33.45 |
| 6 | 26.68 | 26.31 | 26.80 | 26.82 | 27.23 | 28.74 | 31.77 | 32.91 |
| 22 | 20.06 | 20.85 | 21.04 | 21.26 | 21.43 | 21.22 | 26.89 | 30.45 |
|  |  |  |  |  |  |  |  |  |
|  | **gsPCR ΔCt from the 32ug/mL Culture** | | | | | | | |
| gsPCR 4hr | 3.76 | 3.88 | 3.77 | **3.25** | 4.60 | **3.00** | 3.71 | 0.00 |
| gsPCR 6hr | 6.23 | 6.60 | 6.11 | 6.09 | 5.68 | 4.17 | **1.14** | 0.00 |
| gsPCR 22hr | 10.39 | 9.60 | 9.41 | 9.19 | 9.02 | 9.23 | 3.56 | **0.00** |

| **ETGA Ct of Purified MRSA v Concentration of Vancomycin (µg/mL) in Culture Over Time** | | | | | | | | |
| --- | --- | --- | --- | --- | --- | --- | --- | --- |
|  | 0 | 0.25 | 0.5 | 1 | 2 | 4 | 8 | 16 |
| **hours** |  |  |  |  |  |  |  |  |
| 0 | 27.71 | 27.43 | 27.97 | 27.91 | 27.56 | 27.45 | 27.75 | 28.17 |
| 2 | 25.10 | 26.87 | 27.64 | 26.99 | 26.85 | 26.45 | 26.47 | 26.73 |
| 4 | 21.79 | 26.97 | 27.63 | 27.24 | 26.87 | 27.08 | 26.42 | 26.86 |
| 6 | 18.41 | 26.93 | 27.12 | 27.24 | 27.24 | 27.31 | 26.71 | 26.90 |
| 22 | 14.14 | 27.92 | 28.19 | 27.89 | 28.24 | 28.03 | 27.27 | 27.70 |
|  |  |  |  |  |  |  |  |  |
| **ETGA ΔCt from the 16ug/mL Culture** | | | | | | | | |
| 4hr | 0.00 | **5.18** | 5.84 | 5.45 | 5.08 | 5.29 | 4.63 | 5.07 |
| 6hr | 0.00 | **8.52** | 8.71 | 8.83 | 8.83 | 8.90 | 8.30 | 8.49 |
| 22hr | 0.00 | **13.78** | 14.05 | 13.75 | 14.10 | 13.89 | 13.13 | 13.56 |

ETGA Reagent Control Ct: 36.59

Polymerase Control: 27.07

Inoculation Verification of Cultures: 50 CFU

| **gsPCR Ct of Purified MRSA v Concentration of Vancomycin (µg/mL) in Culture Over Time** | | | | | | | | |
| --- | --- | --- | --- | --- | --- | --- | --- | --- |
|  | 0 | 0.25 | 0.5 | 1 | 2 | 4 | 8 | 16 |
| **hours** |  |  |  |  |  |  |  |  |
| 0 | 32.93 | 32.67 | 34.60 | 34.30 | 34.31 | 34.28 | 34.29 | 35.24 |
| 2 | 31.16 | 33.33 | 33.19 | 33.28 | 33.54 | 33.77 | 33.31 | 33.71 |
| 4 | 28.26 | 32.36 | 33.79 | 32.99 | 33.44 | 33.15 | 32.86 | 33.02 |
| 6 | 24.81 | 33.43 | 32.95 | 33.03 | 33.67 | 32.84 | 33.54 | 33.62 |
| 22 | 19.41 | 32.50 | 32.94 | 32.34 | 32.82 | 32.51 | 32.13 | 32.44 |
|  |  |  |  |  |  |  |  |  |
| **gsPCR ΔCt from the 16ug/mL Culture** | | | | | | | | |
| 4hr | 4.76 | **0.66** | -0.77 | 0.03 | -0.42 | -0.13 | 0.16 | 0.00 |
| 6hr | 8.81 | **0.19** | 0.67 | 0.59 | -0.05 | 0.78 | 0.08 | 0.00 |
| 22hr | 13.03 | **-0.06** | -0.50 | 0.10 | -0.38 | -0.07 | 0.31 | 0.00 |

| **ETGA Ct of Purified *E. coli* v Concentration of Ciprofloxacin (µg/mL) in Culture Over Time** | | | | | | | | |
| --- | --- | --- | --- | --- | --- | --- | --- | --- |
| Hours | 0 | 0.002 | 0.004 | 0.008 | 0.016 | 0.031 | 0.063 | 0.125 |
| 0 | 28.54 | 27.60 | 28.20 | 27.47 | 27.28 | 28.15 | 27.78 | 27.51 |
| 2 | 27.28 | 27.75 | 26.85 | 28.33 | 27.75 | 28.73 | 30.14 | 30.70 |
| 4 | 19.80 | 21.76 | 25.10 | 26.64 | 26.86 | 27.37 | 27.32 | 27.45 |
| 6 | 17.32 | 18.91 | 24.05 | 26.88 | 27.02 | 26.87 | 27.27 | 27.46 |
| 22 | 14.34 | 14.71 | 16.67 | 27.67 | 28.16 | 28.64 | 26.91 | 27.06 |
| **ETGA ΔCt from the 0.125ug/mL Culture** | | | | | | | | |
|  | 0 | 0.002 | 0.004 | 0.008 | 0.016 | 0.031 | 0.063 | 0.125 |
| Δ4 hrs | 7.65 | 5.69 | **2.35** | 1.45 | 0.59 | 0.08 | 0.13 | 0.00 |
| Δ6 hrs | 10.14 | 8.55 | 3.41 | **0.58** | 0.44 | 0.59 | 0.19 | 0.00 |
| Δ22 hrs | 12.72 | 12.35 | 10.39 | **-0.61** | -1.58 | -1.10 | 0.15 | 0.00 |

ETGA Reagent Control Ct: 33.60

Polymerase Control: 27.54

Inoculation Verification of Cultures: 65 CFU

| **gsPCR Ct of Purified *E. coli* v Concentration of Ciprofloxacin (µg/mL) in Culture Over Time** | | | | | | | | |
| --- | --- | --- | --- | --- | --- | --- | --- | --- |
| Hours | 0 | 0.002 | 0.004 | 0.008 | 0.016 | 0.031 | 0.063 | 0.125 |
| 0 | 37.45 | 35.23 | 37.11 | 34.75 | 35.87 | 36.86 | 35.54 | 34.25 |
| 2 | 32.62 | 33.78 | 33.43 | 36.13 | 34.56 | 34.99 | 35.58 | 35.49 |
| 4 | 26.96 | 29.57 | 33.91 | 33.95 | 35.03 | 33.91 | 34.77 | 35.18 |
| 6 | 23.85 | 27.01 | 32.61 | 34.77 | 34.36 | 34.48 | 34.64 | 36.68 |
| 22 | 22.55 | 22.96 | 23.83 | 34.86 | 34.82 | 35.99 | 35.03 | 36.21 |
| **gsPCR ΔCt from the 0.125ug/mL Culture** | | | | | | | | |
|  | 0 | 0.002 | 0.004 | 0.008 | 0.016 | 0.031 | 0.063 | 0.125 |
| Δ4 hrs | 8.22 | 5.61 | **1.27** | 1.23 | 0.15 | 1.27 | 0.41 | 0.00 |
| Δ6 hrs | 12.83 | 9.67 | 4.07 | **1.91** | 2.32 | 2.20 | 2.04 | 0.00 |
| Δ22 hrs | 13.66 | 13.25 | 12.38 | **1.35** | 1.39 | 0.22 | 1.18 | 0.00 |

| **ETGA Ct of Purified *E. coli* v Concentration of Tetracycline in Culture Over Time** | | | | | | | | |
| --- | --- | --- | --- | --- | --- | --- | --- | --- |
|  | 0 | 0.063 | 0.125 | 0.25 | 0.5 | 1 | 2 | 4 |
| **hours** |  |  |  |  |  |  |  |  |
| 0 | 26.67 | 26.76 | 26.66 | 27.17 | 26.60 | 26.71 | 26.57 | 27.24 |
| 2 | 23.88 | 23.46 | 24.04 | 25.30 | 25.77 | 26.57 | 26.21 | 26.94 |
| 4 | 18.57 | 18.38 | 18.53 | 19.79 | 23.91 | 25.81 | 27.00 | 27.01 |
| 6 | 16.04 | 16.47 | 16.77 | 17.94 | 23.59 | 26.08 | 26.08 | 26.79 |
| 22 | 13.84 | 13.88 | 13.74 | 14.08 | 13.19 | 24.09 | 26.91 | 26.62 |
|  |  |  |  |  |  |  |  |  |
| **ETGA ΔCt from the 4ug/mL Culture** | | | | | | | | |
| 4hr | 8.44 | 8.63 | 8.48 | 7.22 | **3.10** | 1.20 | 0.01 | 0.00 |
| 6hr | 10.75 | 10.32 | 10.02 | 8.85 | **3.20** | 0.71 | 0.71 | 0.00 |
| 22hr | 12.78 | 12.74 | 12.88 | 12.54 | 13.43 | **2.53** | -0.29 | 0.00 |

ETGA Reagent Control Ct: 36.44

Polymerase Control: 27.47

Inoculation Verification of Cultures: 28 CFU

| **gsPCR Ct of Purified *E. coli* v Concentration of Tetracycline (µg/mL) in Culture Over Time** | | | | | | | | |
| --- | --- | --- | --- | --- | --- | --- | --- | --- |
|  | 0 | 0.063 | 0.125 | 0.25 | 0.5 | 1 | 2 | 4 |
| **hours** |  |  |  |  |  |  |  |  |
| 0 | 34.88 | 36.01 | 36.09 | 36.45 | 36.22 | 35.95 | 36.11 | 37.04 |
| 2 | 34.81 | 33.63 | 34.71 | 35.57 | 35.04 | 34.18 | 33.79 | 35.32 |
| 4 | 29.08 | 28.28 | 27.64 | 28.64 | 30.24 | 32.23 | 34.22 | 34.18 |
| 6 | 26.49 | 25.08 | 26.83 | 27.71 | 31.28 | 33.28 | 34.79 | 35.63 |
| 22 | 16.49 | 15.08 | 16.83 | 17.71 | 21.28 | 30.72 | 33.74 | 32.86 |
|  |  |  |  |  |  |  |  |  |
| **gsPCR ΔCt from the 4ug/mL Culture** | | | | | | | | |
| 4hr | 5.10 | 5.90 | 6.54 | 5.54 | 3.94 | **1.95** | -0.04 | 0.00 |
| 6hr | 9.14 | 10.55 | 8.80 | 7.92 | 4.35 | **2.35** | 0.84 | 0.00 |
| 22hr | 16.37 | 17.78 | 16.03 | 15.15 | 11.58 | **2.14** | -0.88 | 0.00 |

**Supplemental Data Set 2:** ETGA and gsPCR Ct Data of AST Experiments from Cultures Harvested from Positive Blood Cultures. Values in bold indicate the concentration in which the MIC was called. Values in red indicate discrepancies in the results.

| **ETGA Ct of BC Harvested MSSA v Concentration of Oxacillin (µg/mL) in Culture Over Time** | | | | | | | | |
| --- | --- | --- | --- | --- | --- | --- | --- | --- |
| hour | 0 | 0.125 | 0.25 | 0.5 | 1 | 2 | 4 | 8 |
| 0 | 29.63 | 29.69 | 28.83 | 29.08 | 30.68 | 29.03 | 30.02 | 29.35 |
| 2 | 26.49 | 25.92 | 26.75 | 26.66 | 27.22 | 27.50 | 28.29 | 28.52 |
| 4 | 22.12 | 23.55 | 25.70 | 27.74 | 27.81 | 28.17 | 28.28 | 27.94 |
| 6 | 19.62 | 22.95 | 26.45 | 27.92 | 29.50 | 28.68 | 28.70 | 29.25 |
|  |  |  |  |  |  |  |  |  |
| **ETGA ΔCt from the 8ug/mL Culture** | | | | | | | | |
| 4hr | 5.82 | 4.39 | **2.24** | 0.20 | 0.13 | -0.23 | -0.34 | 0.00 |
| 6hr | 9.63 | 6.30 | **2.80** | 1.33 | -0.25 | 0.57 | 0.55 | 0.00 |

ETGA Reagent Control Ct: 34.49

Polymerase Control: 27.43

Inoculation Verification of Cultures: 0 CFU (too low to count)

| **gsPCR Ct of BC Harvested MSSA v Concentration of Oxacillin (µg/mL) in Culture Over Time** | | | | | | | | |
| --- | --- | --- | --- | --- | --- | --- | --- | --- |
| hour | 0 | 0.125 | 0.25 | 0.5 | 1 | 2 | 4 | 8 |
| 0 | 36.65 | 35.72 | 35.81 | 36.66 | 36.94 | 36.16 | 36.98 | 36.77 |
| 2 | 33.65 | 32.96 | 34.57 | 33.87 | 34.52 | 34.67 | 35.78 | 35.04 |
| 4 | 29.49 | 30.80 | 32.56 | 33.80 | 34.24 | 34.95 | 34.68 | 34.45 |
| 6 | 26.27 | 29.25 | 31.83 | 33.71 | 33.65 | 34.29 | 33.87 | 33.28 |
|  |  |  |  |  |  |  |  |  |
| **gsPCR ΔCt from 8ug/mL of Oxacillin** | | | | | | | | |
| 4hr | 4.96 | 3.65 | **1.89** | 0.65 | 0.21 | -0.50 | -0.23 | 0.00 |
| 6hr | 7.01 | 4.03 | **1.45** | -0.43 | -0.37 | -1.01 | -0.59 | 0.00 |

| **ETGA Ct of BC Harvested MSSA v Concentration of Vancomycin (µg/mL) in Culture Over Time** | | | | | | | | |
| --- | --- | --- | --- | --- | --- | --- | --- | --- |
| hour | 0 | 0.125 | 0.25 | 0.5 | 1 | 2 | 4 | 8 |
| 0 | 29.07 | 29.53 | 27.22 | 30.07 | 28.78 | 28.66 | 28.54 | 28.28 |
| 2 | 26.11 | 29.50 | 28.71 | 27.63 | 28.19 | 29.27 | 28.82 | 28.01 |
| 4 | 23.31 | 29.09 | 30.57 | 30.56 | 30.22 | 27.31 | 29.63 | 29.29 |
| 6 | 19.67 | 29.72 | 30.53 | 29.96 | 30.67 | 29.07 | 26.52 | 28.58 |
|  |  |  |  |  |  |  |  |  |
| **PolMA ΔCt from 8ug/mL of Vancomycin** | | | | | | | | |
| 4hr | 5.98 | **0.20** | -1.28 | -1.27 | -0.93 | 1.98 | -0.34 | 0.00 |
| 6hr | 8.91 | **-1.14** | -1.95 | -1.38 | -2.09 | -0.49 | 2.06 | 0.00 |

ETGA Reagent Control Ct: 34.49

Polymerase Control: 27.43

Inoculation Verification of Cultures: 1 CFU

| **gsPCR Ct of BC Harvested MSSA v Concentration of Vancomycin (µg/mL) in Culture Over Time** | | | | | | | | |
| --- | --- | --- | --- | --- | --- | --- | --- | --- |
| hour | 0 | 0.125 | 0.25 | 0.5 | 1 | 2 | 4 | 8 |
| 0 | 37.45 | 37.01 | 35.49 | 37.19 | 35.45 | 36.11 | 34.86 | 35.26 |
| 2 | 33.97 | 36.73 | 36.69 | 35.67 | 34.80 | 34.93 | 35.12 | 34.62 |
| 4 | 29.60 | 35.53 | 37.30 | 37.23 | 36.72 | 34.74 | 36.53 | 35.24 |
| 6 | 26.79 | 36.18 | 36.81 | 36.16 | 35.46 | 35.09 | 33.42 | 35.28 |
|  |  |  |  |  |  |  |  |  |
|  | **gsPCR ΔCt from 8ug/mL of Vancomycin** | | | | | | | |
| 4hr | 5.64 | **-0.29** | -2.06 | -1.99 | -1.48 | 0.50 | -1.29 | 0.00 |
| 6hr | 8.49 | **-0.90** | -1.53 | -0.88 | -0.18 | 0.19 | 1.86 | 0.00 |

| **ETGA Ct of BC Harvested MRSA v Concentration of Oxacillin (µg/mL) in Culture Over Time** | | | | | | | | |
| --- | --- | --- | --- | --- | --- | --- | --- | --- |
| hour | 0 | 0.5 | 1 | 2 | 4 | 8 | 16 | 32 |
| 0 | 31.06 | 32.46 | 31.67 | 32.93 | 30.96 | 33.71 | 30.78 | 34.49 |
| 2 | 30.51 | 30.19 | 28.92 | 30.59 | 30.61 | 32.13 | 32.12 | 34.73 |
| 4 | 25.94 | 25.65 | 26.06 | 27.98 | 27.34 | 32.69 | 31.02 | 34.24 |
| 6 | 22.60 | 23.80 | 24.82 | 26.21 | 26.45 | 27.94 | 32.45 | 31.84 |
|  |  |  |  |  |  |  |  |  |
| **gsPCR ΔCt from 32ug/mL of Oxacillin** | | | | | | | | |
| 4hr | 8.30 | 8.59 | 8.18 | 6.26 | 6.90 | **1.55** | 3.22 | 0.00 |
| 6hr | 9.24 | 8.04 | 7.02 | 5.63 | 5.39 | 3.90 | **-0.61** | 0.00 |

ETGA Reagent Control Ct: 34.78

Polymerase Control: 11.69 (signal produced from undiluted polymerase stock)

Inoculation Verification of Cultures: 0 CFU (too low to count)

| **gsPCR Ct of BC Harvested MRSA v Concentration of Oxacillin (ug/mL) in Culture Over Time** | | | | | | | | |
| --- | --- | --- | --- | --- | --- | --- | --- | --- |
| hour | 0 | 0.5 | 1 | 2 | 4 | 8 | 16 | 32 |
| 0 | 37.15 | 38.00 | 37.38 | 37.85 | 40.00 | 38.00 | 38.19 | 38.00 |
| 2 | 38.00 | 36.70 | 35.80 | 37.30 | 36.73 | 38.00 | 36.66 | 38.00 |
| 4 | 33.46 | 33.55 | 33.11 | 34.29 | 33.96 | 35.23 | 35.99 | 38.00 |
| 6 | 29.69 | 30.58 | 28.58 | 33.06 | 32.90 | 33.29 | 36.73 | 36.17 |
|  |  |  |  |  |  |  |  |  |
| **gsPCR ΔCt from 32ug/mL of Oxacillin** | | | | | | | | |
| 4hr | 4.54 | 4.45 | 4.89 | 3.71 | 4.04 | **2.77** | 2.01 | 0.00 |
| 6hr | 6.48 | 5.59 | 7.59 | **3.11** | 3.27 | 2.88 | -0.56 | 0.00 |

| **ETGA Ct of BC Harvested MRSA v Concentration of Vancomycin (µg/mL) in Culture Over Time** | | | | | | | | |
| --- | --- | --- | --- | --- | --- | --- | --- | --- |
| hour | 0 | 0.5 | 1 | 2 | 4 | 8 | 16 | 32 |
| 0 | 31.91 | 31.74 | 31.93 | 32.85 | 32.34 | 30.55 | 31.88 | 31.80 |
| 2 | 30.13 | 34.80 | 35.88 | 35.37 | 31.27 | 32.31 | 31.77 | 31.99 |
| 4 | 27.01 | 30.75 | 35.22 | 32.59 | 34.64 | 33.58 | 27.03 | 30.79 |
| 6 | 23.70 | 32.25 | 32.58 | 35.08 | 34.63 | 34.49 | 31.72 | 32.61 |
|  |  |  |  |  |  |  |  |  |
|  | **ETGA ΔCt from 32ug/mL of Vancomycin** | | | | | | | |
| 4hr | 3.78 | **0.04** | -4.43 | -1.80 | -3.85 | -2.79 | **3.76** | 0.00 |
| 6hr | 8.91 | **0.36** | 0.03 | -2.47 | -2.02 | -1.88 | 0.89 | 0.00 |

ETGA Reagent Control Ct: 34.78

Polymerase Control: 11.69 (signal produced from undiluted polymerase stock)

Inoculation Verification of Cultures: 0 CFU (too low to count)

| **gs-PCR Ct of BC Harvested MRSA v Concentration of Vancomycin (µg/mL) in Culture Over Time*** | | | | | | | | |
| --- | --- | --- | --- | --- | --- | --- | --- | --- |
| hour | 0 | 0.5 | 1 | 2 | 4 | 8 | 16 | 32 |
| 0 | 38.00 | 36.57 | 38.00 | 38.00 | 38.00 | 35.87 | 38.00 | 36.18 |
| 2 | 37.39 | 38.00 | 37.83 | 38.00 | 37.25 | 36.90 | 38.00 | 35.51 |
| 4 | 35.02 | 37.43 | 38.00 | 38.00 | 38.00 | 38.00 | 38.00 | 37.02 |
| 6 | 30.73 | 36.87 | 37.12 | 36.98 | 38.00 | 38.00 | 36.88 | 36.31 |
|  |  |  |  |  |  |  |  |  |
| **gsPCR ΔCt from 32ug/mL of Vancomycin** | | | | | | | | |
| 4hr | 2.00 | **-0.41** | -0.98 | -0.98 | -0.98 | -0.98 | -0.98 | 0.00 |
| 6hr | 5.58 | **-0.56** | -0.81 | -0.67 | -1.69 | -1.69 | -0.57 | 0.00 |

*For the gene-specific PCR data, many reactions were not detected. This negative values were most likely due to the true inoculation of the cultures being lower than 5E+05 CFU/mL, despite the fact that inoculation of the cultures were based on a 0.5 unit McFarland bacterial suspension. These negative reactions were given an arbitrary Ct value of 38.00, the approximate cycle in which a single copy a gene target is detected, in order to establish a baseline.

| **ETGA Ct of BC Harvested *E. coli* v Concentration of Ciprofloxacin in Culture Over Time** | | | | | | | | |
| --- | --- | --- | --- | --- | --- | --- | --- | --- |
| **hours** | 0.000 | 0.002 | 0.004 | 0.008 | 0.016 | 0.031 | 0.063 | 0.125 |
| 0 | 28.68 | 29.04 | 28.75 | 28.92 | 28.74 | 28.91 | 29.01 | 28.95 |
| 2 | 25.27 | 24.68 | 25.33 | 25.53 | 25.81 | 27.33 | 28.25 | 28.45 |
| 4 | 20.50 | 21.14 | 22.86 | 26.36 | 27.60 | 28.26 | 28.67 | 28.84 |
| 6 | 16.87 | 17.54 | 21.99 | 26.03 | 27.07 | 28.63 | 28.08 | 28.61 |
|  |  |  |  |  |  |  |  |  |
| **ETGA ΔCt from 0.125ug/mL of Ciprofloxacin** | | | | | | | | |
|  | 0 | 0.002 | 0.004 | 0.008 | 0.016 | 0.031 | 0.063 | 0.125 |
| 4hr | 8.34 | 7.70 | 5.98 | **2.48** | 1.24 | 0.58 | 0.17 | 0.00 |
| 6hr | 11.74 | 10.54 | 6.62 | **2.58** | 1.54 | -0.02 | 0.53 | 0.00 |

ETGA Reagent Control Ct: 36.08.

Polymerase Control: 26.78.

Inoculation Verification of Cultures: 15 CFU

| **gsPCR Ct of BC Harvested *E. coli* v Concentration of Ciprofloxacin in Culture Over Time** | | | | | | | | |
| --- | --- | --- | --- | --- | --- | --- | --- | --- |
| **hours** | 0.000 | 0.002 | 0.004 | 0.008 | 0.016 | 0.031 | 0.063 | 0.125 |
| 0 | 36.00 | 35.81 | 35.89 | 37.32 | 35.32 | 35.08 | 35.43 | 36.15 |
| 2 | 33.71 | 34.93 | 34.77 | 35.73 | 34.88 | 34.53 | 33.86 | 34.61 |
| 4 | 28.97 | 30.19 | 31.87 | 34.14 | 35.49 | 34.20 | 35.18 | 34.68 |
| 6 | 24.76 | 26.25 | 30.14 | 33.45 | 33.20 | 34.70 | 34.41 | 34.48 |
|  |  |  |  |  |  |  |  |  |
| **gsPCR ΔCt from 0.125ug/mL of Ciprofloxacin** | | | | | | | | |
|  |  |  |  |  |  |  |  |  |
| 4hr | 5.71 | 4.49 | **2.81** | 0.54 | -0.81 | 0.48 | -0.50 | 0.00 |
| 6hr | 9.72 | 8.23 | 4.34 | **1.03** | 1.28 | -0.22 | 0.07 | 0.00 |

| **ETGA Ct of BC Harvested *E. coli* v Concentration (µg/mL) of Tetracycline in Culture Over Time** | | | | | | | | |
| --- | --- | --- | --- | --- | --- | --- | --- | --- |
| **hours** | 0 | 0.063 | 0.125 | 0.25 | 0.5 | 1 | 2 | 4 |
| 0 | 28.91 | 28.60 | 28.68 | 28.93 | 28.97 | 29.29 | 29.06 | 29.75 |
| 2 | 24.72 | 25.18 | 25.72 | 27.59 | 29.01 | 28.69 | 28.70 | 29.11 |
| 4 | 19.80 | 20.51 | 20.99 | 24.56 | 27.36 | 28.80 | 28.76 | 28.95 |
| 6 | 16.90 | 16.83 | 17.46 | 24.77 | 25.99 | 28.47 | 28.49 | 29.07 |
|  |  |  |  |  |  |  |  |  |
| **ETGA ΔCt from 4/mL of Tetracycline** | | | | | | | | |
|  | 0 | 0.063 | 0.125 | 0.25 | 0.5 | 1 | 2 | 4 |
| 4hr | 9.15 | 8.44 | 7.96 | 4.39 | **1.59** | 0.15 | 0.19 | 0.00 |
| 6hr | 12.17 | 12.24 | 11.61 | 4.30 | **3.08** | 0.60 | 0.58 | 0.00 |

ETGA Reagent Control Ct: 36.08.

Polymerase Control: 26.78.

Inoculation Verification of Cultures: 24 CFU

| **gsPCR Ct of BC Harvested *E. coli* v Concentration (µg/ml) of Tetracycline in Culture Over Time** | | | | | | | | |
| --- | --- | --- | --- | --- | --- | --- | --- | --- |
| **hours** | 0 | 0.063 | 0.125 | 0.25 | 0.5 | 1 | 2 | 4 |
| 0 | 35.95 | 35.57 | 36.48 | 35.94 | 35.31 | 34.52 | 33.72 | 36.21 |
| 2 | 34.32 | 35.36 | 34.88 | 35.55 | 35.62 | 34.39 | 35.38 | 35.82 |
| 4 | 29.08 | 29.79 | 30.53 | 33.96 | 33.03 | 34.94 | 34.73 | 34.84 |
| 6 | 25.04 | 26.25 | 26.72 | 28.31 | 31.67 | 33.74 | 35.61 | 34.72 |
|  |  |  |  |  |  |  |  |  |
| **gsPCR ΔCt from 4/mL of Tetracycline** | | | | | | | | |
|  | 0 | 0.063 | 0.125 | 0.25 | 0.5 | 1 | 2 | 4 |
| 4hr | 5.76 | 5.05 | 4.31 | **0.88** | 1.81 | -0.10 | 0.11 | 0.00 |
| 6hr | 9.68 | 8.47 | 8.00 | 6.41 | **3.05** | 0.98 | -0.89 | 0.00 |
